# Relationships between genomic dissipation and *de novo* SNP evolution

**DOI:** 10.1371/journal.pone.0303257

**Published:** 2024-05-16

**Authors:** Zackery E. Plyler, Christopher W. McAtee, Aubrey E. Hill, Michael R. Crowley, Janice M. Tindall, Samuel R. Tindall, Disha Joshi, Eric J. Sorscher

**Affiliations:** 1 Department of Biology, University of Alabama at Birmingham, Birmingham, Alabama, United States of America; 2 Gregory Fleming James Cystic Fibrosis Research Center, University of Alabama at Birmingham, Birmingham, Alabama, United States of America; 3 Department of Computer and Information Sciences, University of Alabama at Birmingham, Birmingham, Alabama, United States of America; 4 Department of Genetics, University of Alabama at Birmingham, Birmingham, Alabama, United States of America; 5 Emory University, Atlanta, Georgia, United States of America; CNR, ITALY

## Abstract

Patterns of single nucleotide polymorphisms (SNPs) in eukaryotic DNA are traditionally attributed to selective pressure, drift, identity descent, or related factors—without accounting for ways in which bias during *de novo* SNP formation, itself, might contribute. A functional and phenotypic analysis based on evolutionary resilience of DNA points to decreased numbers of non-synonymous SNPs in human and other genomes, with a predominant component of SNP depletion in the human gene pool caused by robust preferences during *de novo* SNP formation (rather than selective constraint). Ramifications of these findings are broad, belie a number of concepts regarding human evolution, and point to a novel interpretation of evolving DNA across diverse species.

## Introduction

### Relationships between eukaryotic mutation rate and DNA attrition

#### ‘Mutational meltdown’ and DNA evolution

The possibility that SNP accrual over an evolutionary time frame would abolish integrity of genomic DNA was debated decades ago, including cumulative effects of deleterious alleles (so-called ‘meltdown’). For example, it can be argued that at random mutational rates exceeding a certain high level of μ (mutations /genome / generation) in any species, meltdown becomes inescapable. That perspective implies each new generation is subject to a steadily increasing SNP burden that will not be resolved simply by removing unfit organisms. Beyond a sufficient value of μ, the argument runs, even if specific individuals (or entire species) are expunged by natural selection, the “weeding out” process would fail to prevent accumulating SNPs among all surviving relatives and their descendants. At very high μ, DNA recombination (which traditionally has been suggested as anodyne to genomic meltdown) would fail to surmount accrual of deleterious SNPs. Over hundreds of millions of years—and beyond a specific threshold for μ—no gene would possess an intact sequence suitable to recombine (or reconvert) and reestablish the proper protein coding instructions essential to life [1, 2]. But how large a SNP accumulation rate is *too* large?

#### Evolutionary strategies to avert genomic attrition

**Fig 1:**
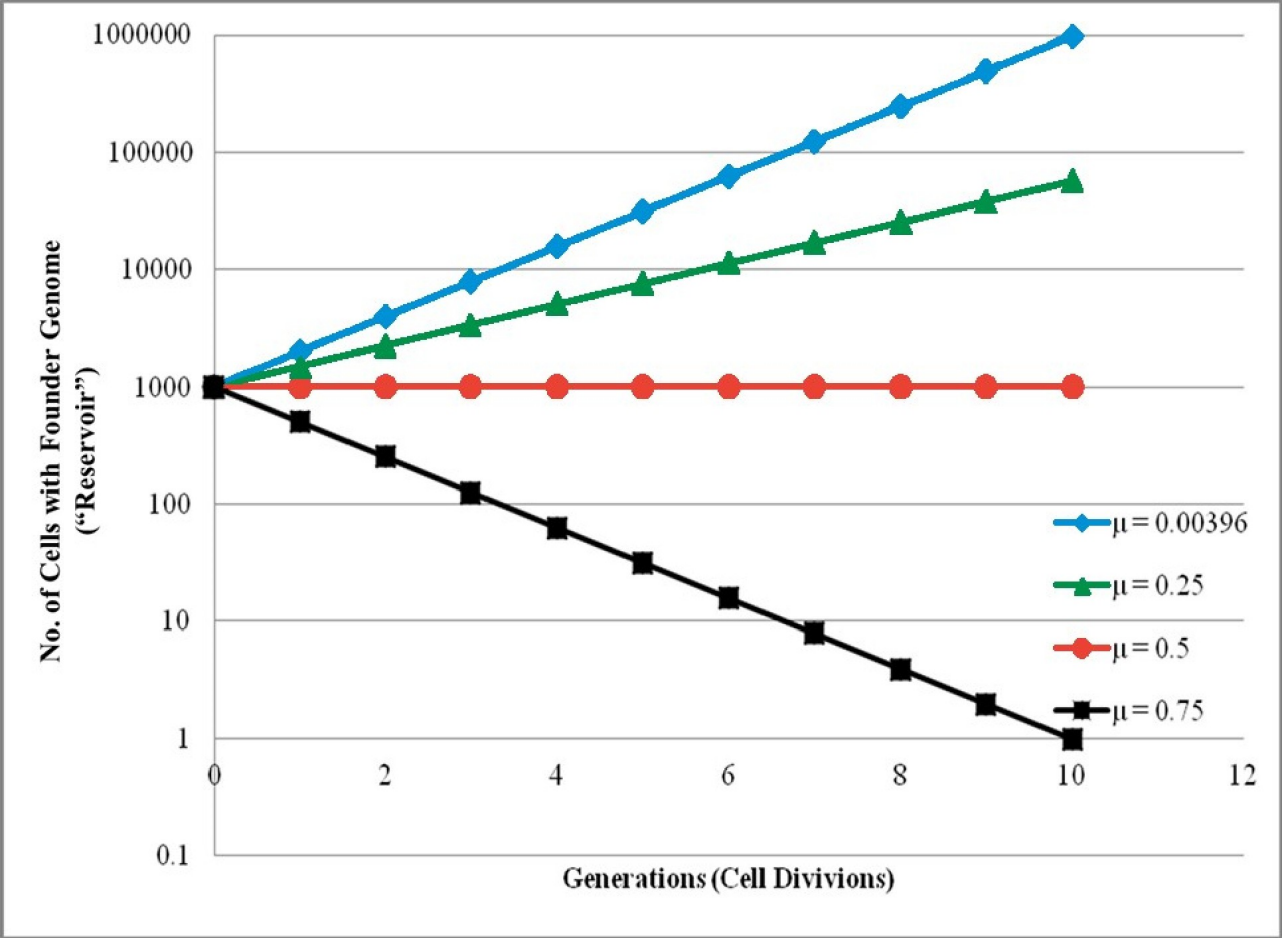
Preservation of the input genome (reservoir) and relationship of DNA meltdown to mutation rate (μ). The number of organisms (from starting population of 1000) retaining a founder genome was modeled at various mutation rates (μ; mutations/genome/generation). When μ = 0 (no new mutations occur), the number of organisms identical to founder represents total population size. Essentially no difference exists in the number of individuals identical to the genetic founder at any generation when μ = 0.5. The reported yeast mutation rate in *S*. *cerevisiae* (0.004 mutations/genome/generation [3]) yields a constantly increasing number of individual yeast that have no tendency towards meltdown based on complete preservation of the original genome. (Note that for μ < 0.5, meltdown in yeast would not occur regardless of asexual growth rate and whether new SNPs increase or diminish yeast proliferation).

Simply put, the tendency towards DNA meltdown can be approached by a “worst case” scenario dependent on μ (Fig 1). At less than 0.5 mutations/genome/generation, significant numbers of organisms retain DNA identical to the founder individual—providing a ‘reservoir’ (i.e., matching copy) of the original (founder) DNA. In that simplified model, the total number of new deleterious or advantageous mutations in a population becomes less relevant: genomic meltdown is precluded without the need to invoke features such as purifying selection, recombination, large cohort size, drift, etc., since a genetic reservoir (identical to DNA in the previous generation) is maintained. For μ in yeast (~0.004 mutations/genome/generation; i.e., ~1 *de novo* SNP in 250 cell divisions [3], similar to a prokaryotic value [4]), DNA meltdown would not occur barring a marked increase in mutation rate.

The situation is quite different for some metazoans (including humans), where significantly larger μ (~50–100 new SNPs per genome per generation [5–9]) might be expected to result in DNA extinction over evolutionary time without countervailing or palliative measures. A case in point is the essential cystic fibrosis transmembrane conductance regulator (*CFTR*) gene. The ancestral *CFTR* has experienced more than 450 million years of (irreversible) mutation accumulation, and even at very conservative estimate of μ over the evolutionary progression of pre-hominid species, meltdown of CFTR (and many other well-characterized ancient DNA loci) might have been expected long ago [1] (see also below).

‘Countervailing mechanisms’ have been described by many groups, although the evolutionary significance has not been well addressed. For example, *de novo* transition SNPs (A↔G or C↔T) are known to be enriched compared to their transversional counterparts in human and most other eukaryotic genomes. Transition SNPs lead to synonymous (as opposed to non-synonymous) mutations. For example, when a new transition SNP occurs at the third position of a codon, ~94% of the resulting amino acid substitutions are synonymous [1, 10–15]. This biased arrangement has rarely been considered as part of evolutionary analysis, but over millennia of SNP accrual, such an effect would serve to maintain integrity of protein coding DNA and help forestall undesired mutational sequelae, including meltdown. As described later, the observed ratios of nonsynonymous to synonymous SNPs would also be influenced by a strong SNP formation bias. As another example of a countervailing mechanism, context-dependent pathways by which SNPs are directed away from protein coding DNA (preserving genomic integrity) and towards non-coding gene regulatory elements (thereby facilitating variation and diversity) are a genome-preserving adaptation reported previously but infrequently considered [2]. Ways in which epigenetic changes in DNA can influence profiles of *de novo* mutations (DNMs) comprise a further protective mechanism that has become increasingly appreciated [16–24]. SNP “non-randomness” in each of these contexts represents an emerging and topical feature of eukaryotic DNA evolution [25–27].

### The mechanism(s) that underlie *de novo* SNP production represent ‘countervailing’ pathways that help prevent meltdown, but conflict with conclusions reached by earlier and ongoing studies of genomic adaptation [1, 2, 16, 17, 25–29]

#### SNP distributions in diverse organisms

In human genes reported by gnomAD [30], the proportion of non-synonymous to synonymous mutations is typically in a range of 1.5–2.5:1. This result indicates non-synonymous SNP depletion—since the ratio of non-synonymous to synonymous SNPs expected on a random basis (from the genetic code corrected for codon usage) is ~3.1:1. A standard and well established data resource, therefore, indicates millions of non-synonymous SNPs are missing from every human genome. In earlier studies among zebrafish, frog, chicken, platypus, opossum, cow, elephant, and chimpanzee, each species exhibited strong non-synonymous SNP depletion genome-wide similar to humans (using a conventional d_N_/d_S_ metric [31]). Non-synonymous SNP depletion of this type is most commonly attributed to deleterious fitness effects (i.e., selective constraint), and has been employed for many years as a standard for distinguishing essential (versus expendable) genetic loci, DNA evolutionary rates, gene adaptation, species divergence/ relatedness, pedigrees, protein sequence conservation, and many other features, based on the reasonable assumption of predominantly random *de novo* mutations. Although a measure of DNM bias has sometimes been acknowledged, strong non-randomness of SNP formation has not been considered by most studies of DNA evolution—and failure to recognize the magnitude and mechanism of biased SNP formation could present a problem. [1, 2, 16, 17, 25–29] and below). For example, recent attempts to identify human loci as “essential” based on non-synonymous to synonymous SNP ratios (available from gnomAD [30]) assume predominately random *de novo* mutations—or at least strong concordance between tabulations of non-synonymous and synonymous DNMs in a particular gene of interest. At present, however, there is insufficient DNM data to validate that assumption (particularly insofar as transition and synonymous DNMs are concerned [9, 15, 25, 26]).

## Results and discussion

### The quantitative extent of bias during *de novo* SNP formation

How stochastic is the process of eukaryotic DNM formation (a primary means of securing adaptive diversity)? To address that question, one may begin by comparing relevant features of SNP production in *S*. *cerevisiae*—including transition/transversion ratios, relationships to protein plasticity/stability, essential versus non-essential genes, intronic mutations, and other evolutionary attributes. *S*. *cerevisiae* was selected so that similarities with other eukaryotes might be investigated and common mechanistic pathways identified. Strong evidence for synonymous and transition SNP enrichment is observed when laboratory strains with a shared ancestor are compared (Tables 1 and 2) [17, 32, 33]. When exonic SNPs are categorized based on observed frequency for classically ‘essential’ versus ‘non-essential’ loci (Table 2) [34, 35], similar non-synonymous to synonymous SNP ratios are noted in both groups—a finding that at face value might suggest against an explanation attributable solely to adaptive pressure or purifying selection (see next section). Corresponding analysis of human [1] and murine[2] genomes have led to conclusions very similar to those shown here for yeast.

**Table 1:** Distribution of validated SNPs in comparison to *S*. *cerevisiae* reference genome (sacCer2).

Region	Total SNPs	Transition	Transversion	Observed Transition / Transversion	Expected Transition / Transversion*
Coding°	1385	907	478	1.897^Δ^	0.500
*Non-synonymous*	677	322	355	0.907^Δ^	0.400σ
*Synonymous*	709	583	126	4.627^Δ^	1.165σ
*Stop Gain*	5	3	2	-	-
*Stop Lost*	3	1	2	-	-
*Synonymous Stop*	1	1	0	-	-
Non-coding	1088	684	404	1.693^Δ^	0.500
*Intronic*	20	13	8	1.625^Δ^	0.500
Totals	2473	1591	882	1.804^Δ^	0.500
*Coding / Noncoding*	1.273^Δ^	-	-	*Expected Coding/Noncoding*	3.000

Expected non-synonymous to synonymous and transition to transversion ratios were calculated from all possible single nucleotide replacements in the genetic code. Expected ratio of coding [36] to non-coding SNPs was based on size of the respective DNA compartments in *S*. *cerevisiae*.

Additional notes

* Transitions (T↔C or A↔G) would otherwise be expected to occur half as frequently compared with transversions (A↔T, A↔C, C↔G, and G↔T), barring natural selection, drift, or features that confer bias in overall incidence.

° Due to overlapping gene sequences (∼3000 overlapping *S*. *cerevisiae* ORFs, Mackiewicz et al., *Nucleic Acids Res*. 1999), 20 SNPs had coding effects on multiple ORFs (e.g., on chr I at position 141032, we observed an A↔T SNP having non-synonymous consequences (TCC↔ ACC; Serine↔Threonine) for the ORF of YAL004W, and also causing a synonymous variant (GGA↔GGT; Glycine) on YAL005C). Overall, we observed 1,385 exonic SNPs altering 1,405 transcript positions.

σ All possible transition and transversion SNPs with incidence corrected for codon usage.

^Δ^p = < 0.05 compared to expected values.

**Table 2:** Non-synonymous: Synonymous and transition: Transversion ratios for exonic SNPs in essential versus non-essential *S*. *cerevisiae* genes.

	All	Essential	Non-Essential	Expected
*Non-synonymous*	677	86	591	-
*Synonymous*	709	101	608	-
*Non-synonymous / Synonymous*	0.955^Δ^	0.851^Δ^	0.972^Δ^	3.174
*Transition*	922	124	798	-
*Transversion*	477	64	413	-
*Transition / Transversion*	1.933^Δ^	1.938^Δ^	1.932^Δ^	0.500

SNPs were analyzed by gene category (essential versus non-essential), SNP consequence (non-synonymous versus synonymous), and SNP type (transition versus transversion). Gene category was independent of SNP consequence (p = 0.446) and type (p = 0.947). The yeast genome contains approximately 5,300 non-essential and 1,300 essential genes [34, 35].

^Δ^p = < 0.05 compared to expected values.

#### How important is natural selection to SNP frequencies observed in *S*. *cerevisiae*?

**Fig 2:**
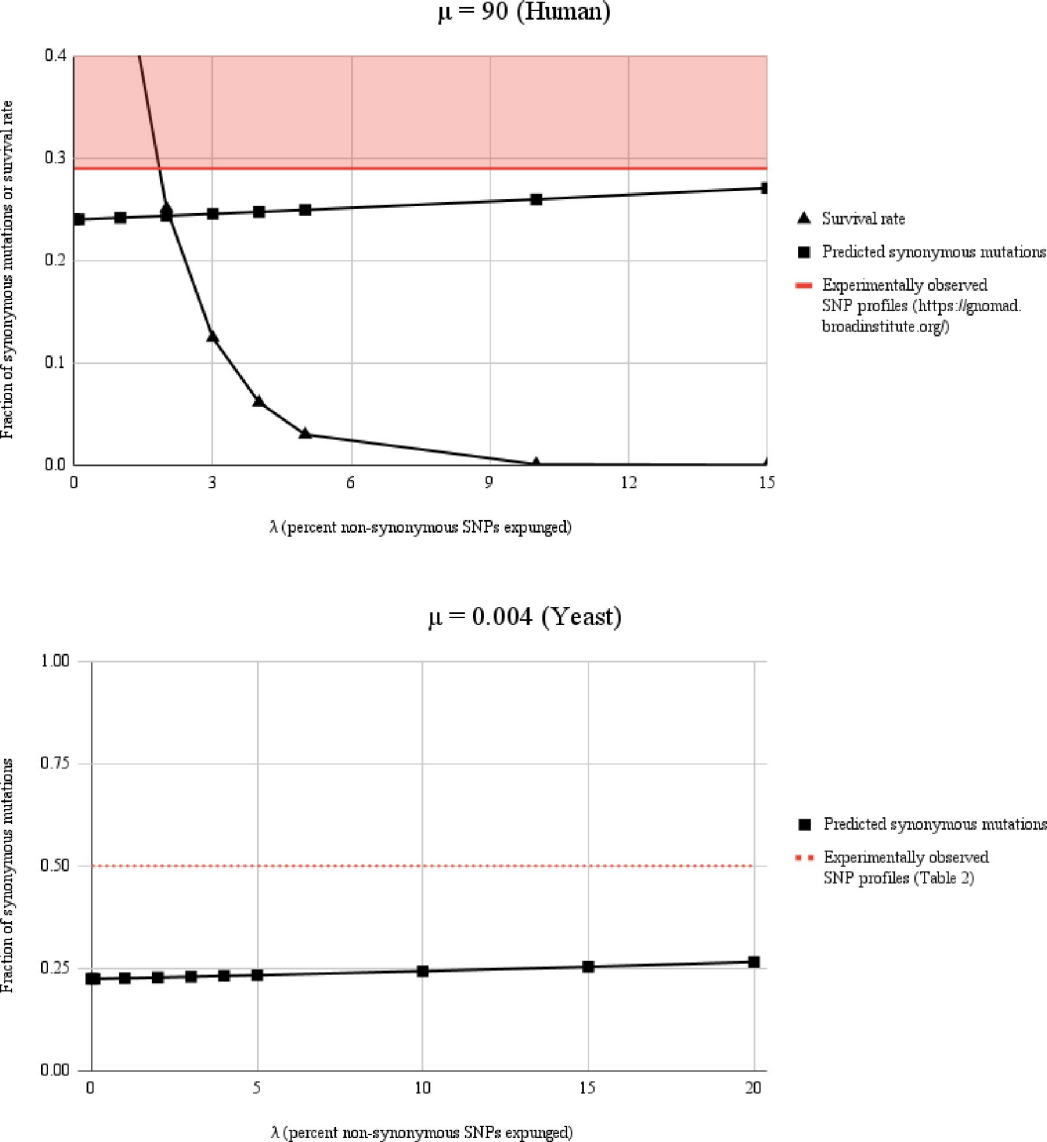
Synonymous mutation frequencies and survival rates at imposed values of μ and λ are in conflict with traditional models of eukaryotic evolution and constraint. Top panel (μ = 90 mutations per genome per generation) At μ observed in *H*. *sapiens*, it is impossible for purifying selection and constraint to account for the synonymous SNP enhancement reported by gnomAD. This is because if non-synonymous SNPs (and their associated genomes) are expunged from the gene pool at rates (λ) necessary to achieve the measured levels of non-synonymous SNP depletion, genomic meltdown occurs extremely rapidly. For any stable λ (i.e., percent non-synonymous SNPs expunged), reaching the experimentally observed level of synonymous SNP enrichment shown by gnomAD is not achievable at μ = 90 based on removal of deleterious genomes (i.e., factors such as strong DNM bias and punctuated evolution must be invoked to explain the observed SNP numbers). Bottom panel (μ = 0.004) At lower μ (as in *S*. *cerevisiae*), even if non-synonymous mutations are removed at very high (and physiologically unrealistic) rates such as λ = 10%, synonymous SNP enhancement cannot come close to achieving non-synonymous to synonymous ratios such as those measured in Table 2 (where the fraction of synonymous mutations is ~0.5). In yeast, because of low μ and a rapidly expanding reservoir, the population escapes meltdown (Fig 1), but cannot achieve the measured fraction of synonymous SNPs based solely on purifying selection (i.e., DNM formation bias and/or punctuated periods of evolution must again be invoked). Red shading and dashed line = experimentally observed values of synonymous SNP frequency obtained from (https://gnomad.broadinstitute.org/ (for human DNA) or Table 2 (for yeast). Data is shown at >10 generations (see also **S1 Text**).

It is important to note that if one invokes adaptive selection to explain SNP frequencies shown in Tables 1 and 2 (i.e., the scientific premise for a large number of constraint-based studies), over 50% of randomly placed non-synonymous mutations must have conferred a very significant (negative) effect on fitness (the expected non-synonymous to synonymous ratio is >3:1, whereas the observed value is close to unity, Table 2). This is in contrast to previous measurements indicating only 0.1%– 2% of randomly placed mutations have a measurably deleterious fitness effect in *S*. *cerevisiae* [6, 37–39]. The notion that 1 of every 2 random, non-synonymous SNPs could disrupt proliferation of an individual yeast organism (with the associated genome lost or undetectable during laboratory growth) also contrasts a modern view of yeast proteins, which accommodate single base replacements much more favorably. The discrepancy is not resolved by considerations such as epistasis, prolonged periods of evolution, or small (cumulative) effects on adaptability. No matter how small the selective pressure, protracted the time period, number of genes involved, etc., the notion that > 50% of random, non-synonymous SNPs would be sufficiently deleterious to eliminate a detectable genome appears unrealistic. For example, as described below, a “weeding out” rate of that magnitude under laboratory culture conditions is incompatible with well understood features such as protein plasticity, genomic stability, published data regarding fitness effects, and similarity of non-synonymous SNP depletion in essential versus non-essential genes (Fig 2, **S1 Text,** and [1, 2, 26, 37–39]). In the following sections, we provide a series of models and carefully framed arguments that support our perspective—and indicate diminished fitness cannot account for the non-synonymous SNP proportions typically observed in yeast by many laboratories. Importantly, genomic DNA findings as shown here are the norm when comparing laboratory yeast strains, laboratory mice [2], and recent human SNPs [15], and may develop over comparatively brief periods of evolution. We believe such findings reflect modest but essential contributions of DNM bias (in combination with effects of selective pressure, punctuated evolution, or other features) that become apparent over time.

#### Relevance of findings in yeast to the human genome

As introduced above, “missing” non-synonymous SNPs are routinely observed in many diploid eukaryotes including humans—which typically exhibit non-synonymous to synonymous ratios of 1.5–2.5:1 (versus the expected value of ~3.1:1 if *de novo* SNPs occur randomly) [30]. An explanation based predominantly on ‘weeding out’ of deleterious polymorphisms from human DNA represents even more of a stretch than in haploid yeast, since a single, random, non-synonymous point mutation in the human genome would be overwhelmingly unlikely, by itself, to cause early death or measurably impair fertility (irrespective of epistasis). It would be naïve, for example, given a modern understanding of mammalian proteins to suggest that any isolated *de novo* non-synonymous SNP placed somewhere randomly (in one of ~20,000 coding genes or > 30 million DNA exonic positions) should have such dramatic effect on fitness that in ~10% of such cases (the difference between expected and observed non-synonymous SNPs in gnomAD), human death or reproductive compromise would result. This is particularly true because of diploidy, where a single point mutation would need to exhibit profound haploinsufficiency to explain such findings (i.e., a functional copy of the same gene is being expressed by the second allele). Once again, even under very extreme conditions—such as heavily inbred populations (congenic laboratory animals maintained for decades in small breeding colonies)—while there is no question that deleterious SNPs are sometimes being removed due to selection (including embryonic mutations that undergo purifying removal prenatally), epistasis or related features should not be overinterpreted to suggest that enormous numbers of *de novo* non-synonymous SNPs are routinely being deleted from the gene pool [1, 15]. Nevertheless, past and current studies of selective constraint have often been predicted on an assumption that *de novo* SNP formation bias has negligible effect on non-synonymous versus synonymous SNP ratios (d_N_/d_S_) in modern genomes [1, 2, 16, 17, 25, 26, 28, 29, 31].

#### Conceptual problems with an assumption of random SNP formation

Here is a simple and perhaps informative example to consider: The gnomAD database establishes that non-synonymous to synonymous SNP ratios across the human genome occur at a ratio of roughly 1.5–2.5 to 1. The expected ratio, based on random SNP accumulation is ~3.1 to 1. In a human exome of >30 million base positions, and across the gene pool, this represents many millions of ‘missing’ non-synonymous SNPs. The traditional and time-honored explanation has been that the absent mutations were deleterious—and have been removed from the ancestral human and pre-hominid gene pools over evolutionary time. A sizable body of work has been (and continues to be) based on that explanation. However, at a value for μ in humans (~90 mutations per genome per generation), the intense level of negative selection required to account for gnomAD findings would mean that a species like ours could not survive. At 90 random mutations per generation—and with ~10% of non-synonymous SNPs (and their associated genomes) being expunged by negative selection—*Homo sapiens* would experience an inescapable risk of meltdown. Note that if human mutations are considered random (the convention and basis for many hundreds of publications based on constraint), at μ = ~100 mutations per generation, on average there should be one new exonic SNP for each reproductive cycle (roughly 1% of the human genome is exonic). By four generations, every individual would encode an average of three new non-synonymous SNPs (the stochastic non-synonymous to synonymous ratio is ~3.1 to 1). By 40 generations, 30 non-synonymous mutations should be present per individual with three of these (10%) expected to be expunged and/or lethal. By 400 generations (~8,000 years), every human genome in this scenario would encode 30 mutations that would be expected to prevent survival or obviate reproduction in order to account for the non-synonymous SNP depletion consistently observed by gnomAD in humans. Once again, the result is independent of small fitness effects or epistasis—which account poorly for the observed ratio of non-synonymous to synonymous SNPs (see Fig 2 and **S1 Text**).

#### Generation of *de novo* DNA polymorphism has evolved to facilitate eukaryotic diversity and help mitigate against meltdown

The quantitative disconnect between stochastic SNP production and observed SNP frequencies in yeast, mouse, and human [1, 2, 15] might suggest key assumptions regarding DNM ‘randomness’ in some cases have been oversimplified, and that important aspects of DNA evolutionary theory (rapidly evolving human genes or genomes, ENCODE, DNA adaptation of malignancy, “evolutionary clocks,” etc.) should be reconsidered. The argument does not mean purifying selection is absent, but instead that the implications of ‘weeding out’ have been overinterpreted across numerous studies and species. In *S*. *cerevisiae*, for example, where μ is much lower than in humans, very high numbers of non-synonymous SNPs are ‘missing’ from a standard laboratory strain (Table 2). The observation cannot be explained by negative selection alone (which could never achieve a measured non-synonymous to synonymous ratio of .955 to 1, in part because of a massively expanding genomic ‘reservoir’) (Table 2, Fig 2, and **S1 Text**). Such findings in yeast and human only make sense if features such as a strong and meaningful DNM bias and/or highly punctuated periods of adaptation are invoked as major contributions to modern SNP tabulations (either of which could undermine many constraint-based conclusions reached previously concerning not only yeast adaptation, but *H*. *sapiens* as well).

#### Massive human genomic analysis shows discordance between non-synonymous to synonymous SNP ratios and predicted loss of function (pLOF) density

For decades, ratios of non-synonymous to synonymous SNPs (typically with d_N_/d_S_ correction, but without considering DNM formation bias) have served as the statistical workhorse to identify open reading frames (or entire genomes) subject to recent selective pressure, rapid evolution, species divergence, etc. The gnomAD repository, for example, includes high stringency exonic DNA sequences from well over 100,000 individuals across multiple ethnicities. One goal of gnomAD has been to identify human genes that rarely exhibit pLOF, as a means to determine ‘essential’ human loci (and exclude proteins at these positions as potential drug targets). The rationale has been that genes with statistically fewer pLOF variants (for example, severe nonsense or splice variants)—in semblance to genes with diminished non-synonymous to synonymous SNP ratios—represent protein coding sequences that poorly tolerate deleterious mutation and are most essential to health.

**Fig 3:**
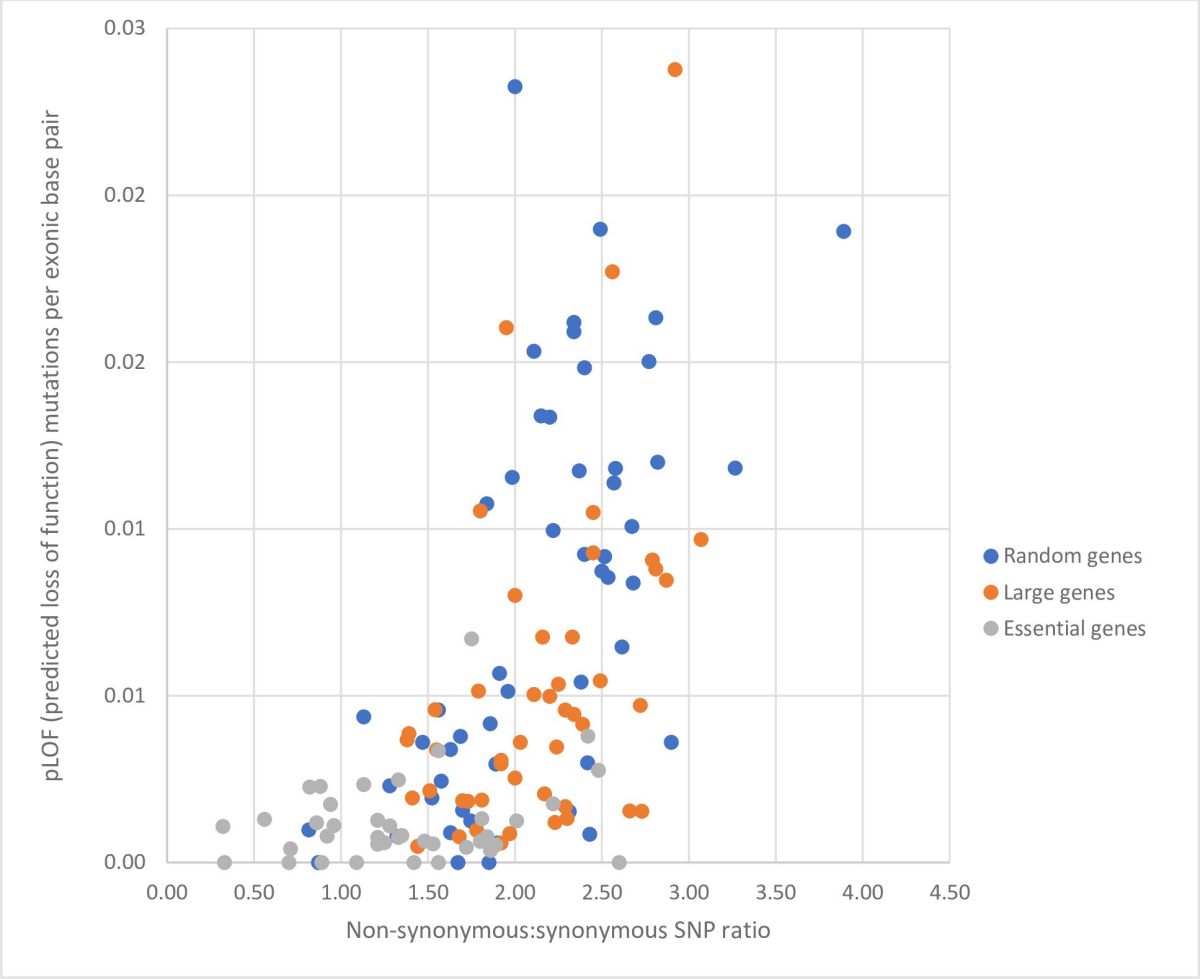
Predicted loss of function (pLOF) variants per exonic base pair versus non-synonymous to synonymous SNP ratio. The gnomAD site (https://gnomad.broadinstitute.org/) was evaluated for 101 randomly selected genes, 47 of the largest genes in the human genome (based on http://www.cshlp.org/ghg5_all/section/gene.shtml [41]), and 140 genes described as essential [40]. Pearson’s correlation coefficient was measured for random genes (r^2^ = 0.3), large genes (r^2^ = 0.2), and essential genes (r^2^ = .06). (See **S1 Text** for additional detail and datasets).

If *de novo* mutations are produced randomly and non-synonymous to synonymous SNP ratios in humans (as judged by gnomAD) truly represent a useful means to track purifying adaptation and selective constraint (i.e., a primary method applied by biologists for decades), strong concordance between low pLOF frequency and non-synonymous SNP depletion might be expected. As shown in Fig 3, this is not the case; i.e., only weak association exists. Moreover, essential genes in humans (identified by population studies, CRISPR-Cas9 cell line deletion, and comparisons with knockout mice [40]) do not exhibit an enhanced correlation of pLOF density when compared to non-synonymous SNP depletion by our analysis (similar to the situation in yeast, Table 2; see also Fig 3, S1 Table in S1 Text). In any case, while certain essential genes with poor haplosufficiency in humans exhibit diminished pLOF counts, many other genes with low pLOF are clearly nonessential, and should not be ruled out as targets for pharmacotherapy.

#### Direct measurement of *de novo* mutations in humans indicate strong SNP non-randomness and question a traditional approach to quantifying *H*. *sapiens* DNA evolution

Several recent human genomic initiatives have provided informative data regarding the ‘randomness’ of *de novo* point mutations. Michaelson et al. studied global and regional ratios of *de novo* SNPs using pairs of monozygotic twins and their parents (i.e., family ‘quad’ analyses) [9]. Dramatic regional variation in SNP formation at specific loci (by up to 100-fold, and with pronounced clustering) was noted, including dependence on features such as chromatin structure and local recombination rate, neither of which has been addressed by evolutionary work based on d_N_/d_S_. Studies from Iossifov et al. indicate an overall synonymous to non-synonymous *de novo* SNP ratio of 30%, depending on the individuals being studied (expected ratio would be ~24% based on random SNP formation corrected for codon usage) [42]. Similarly, in a group of 50 control subjects, O’Roak and colleagues reported a DNM synonymous to non-synonymous ratio of 30–44% [43]. From that perspective, it should be noted that even a small predisposition towards synonymous *de novo* mutations—when present over tens of millions of generations for a particular eukaryotic gene under selective pressure—could have pronounced effects on the SNP frequencies observed today (which otherwise might be misinterpreted as caused solely by intense purifying selection).

Besenbacher et al. investigated 283 parent-offspring trios and identified 17,812 *de novo* single nucleotide mutations [8]. The authors reported significant overrepresentation of mutations in close proximity to each other (non-random SNP formation and pronounced clustering). Importantly, CpG transitions numbered 2984, whereas CpG transversions were 281, consistent with non-random (and marked) enhancement of mutations at CpG sites that would favor *de novo* synonymous (rather than non-synonymous) SNPs. Large numbers of *de novo* transition SNPs (n = 9,022) were unassociated with a CpG motif (i.e., unrelated to classical DNA methylation), and were more likely to occur than non-CpG *de novo* transversions (n = 5,525), despite the fact that on a stochastic basis one would expect roughly twice as many transversions as transitions. Instead, the *de novo* ratio of non-CpG transversion to transition SNPs appears reversed, with mechanisms other than deamination responsible for the resultant enhancement of synonymous point mutations [13, 14]. This data again agrees with the situation in yeast (Tables 1 and 2), where profoundly increased numbers of transition and synonymous SNPs are poorly attributable to DNA methylation (a process very rare in *Saccharomyces cerevisiae*), suggesting a conserved pathway among diverse eukaryotes.

Francioli et al. [44] studied 11,020 *de novo* mutations from 250 families (231 trios, 11 families with monozygotic twins, 8 families with dizygotic twins). DNMs from older fathers were more numerous and occurred frequently in specific DNA regions, with strong evidence of mutational clusters, indicating paternal age dependence and non-randomness of new SNPs. Neale et al. performed whole exome sequencing on 175 autism spectrum disorder trios, and identified exonic DNMs, ~32% of which were synonymous (again, well above the 24% expected on a random basis) [45]. Lek and colleagues performed a large (whole exome) sequencing analysis and found strong evidence for *de novo* mutational recurrence with SNPs repeatedly observed at the same location [46] (see also [30, 47]). In their study, a very large number of validated *de novo* non-synonymous SNPs were compared to DNMs from 1,756 trios, and observed to have occurred more than once. Even greater levels of parallel recurrence (87%) were reported for transition variants at CpG sites (as noted previously in mice, see [2]). Lek also described sequence context influencing highly mutable and less mutable DNA (non-random SNP formation).

While not every report indicates strong divergence from stochastic SNP production, in many cases the magnitude of ‘non-randomness’ would impact a very large body of previous work based on d_N_/d_S_, including studies that have characterized rapidly evolving regions of DNA, genomic divergence of species, pedigree analysis, and other conventional features of genetic epidemiology. Without a clear and quantitative understanding of factors that cause DNM ‘hotspots’ or ‘cold zones,’ inference regarding evolutionary relationships among organisms, rates of gene evolution and divergence, and conserved versus non-conserved regulatory or other loci become much more difficult to interpret. For example, a non-synonymous to synonymous SNP decrement of several-fold (in comparison to the remainder of the human genome) might otherwise be taken to suggest a rapidly evolving DNA domain experiencing selective pressure. However, without accounting for areas of high-level *de novo* SNP formation (at rates augmented 100-fold in certain regions), conclusions such as these need to be reconsidered.

The actual percentages of non-synonymous versus synonymous or coding versus noncoding DNMs are not well defined for individual human genes [15]. This is due to multiple factors such as the extreme rarity of *de novo* mutations, disease context for certain studies (e.g., families with autism), distinguishing germline as opposed to very early somatic mutations leading to purifying selection in the embryo (or long-term chimerism), inability to account for punctuated periods of increased DNM production, epigenetic change in response to environmental stress [16–24], etc. Although our capacity to discriminate DNA bias is not complete, it is clear that even a minimal synonymous and/or non-synonymous DNM preference in a given gene over evolutionary time (thousands or millions of generations) could confuse classical interpretation regarding presence or absence of selective pressure. This feature of genomic evolution has largely been omitted from evolutionary studies in the past because data on topic was unavailable until recently, and DNM findings have not been considered from the perspective of constraint. Although a few reports have attempted to partially account for gene-specific DNM rates and aspects that skew newly formed SNPs [30, 44, 46], considerably more work is needed.

## Conclusions

Based on past experience regarding evolution of ancient human genes (such as CFTR [1, 2, 15, 48]), one question posed by this report involves the tendency towards mutational meltdown, and adaptive measures that forestall destruction of protein coding DNA. Among numerous mechanisms, SNP formation bias has been well demonstrated and is likely to blunt genomic attrition, but has been largely omitted—and primarily viewed as noncontributory from an evolutionary perspective—until recently. The topic requires critical (and quantitative) scrutiny, particularly since a significant body of previous work (and studies continuing at present) have relied on an assumption of ‘random’ SNP formation. The possibility of strikingly non-random *de novo* mutations should be considered in all genomes, including *Homo sapiens*. Such a viewpoint has broad implications, one of which involves preventing dissipation of essential DNA in eukaryotes (for additional examples, see **S1 Text**). Our review of non-synonymous SNP depletion—together with the limitations of evolutionary selection and constraint to adequately account for such findings—suggests conservation of mechanism and a means by which the causal pathways can be better understood in the future.

## Supporting information

S1 Text Supporting information. Includes pLOF frequency in essential versus random or ultra-large genes, a discussion of broad implications of evolutionary mechanisms, including species expansion during the Cambrian and estimating genomic evolution in other settings, such as SARS-CoV-2. Also included are methods used.
(DOCX)

S1 Table Human gene datasets. (XLSX)
