# Supplementary material for: Relationships between genomic dissipation and de novo SNP evolution
Source: PLoS One. 2024 May 16;19(5):e0303257. doi: 10.1371/journal.pone.0303257 (PMC11098520; doi:10.1371/journal.pone.0303257)
Supplement: S1 Text — Includes pLOF frequency in essential versus random or ultra-large genes, a discussion of broad implications of evolutionary mechanisms, including species expansion during the Cambrian and estimating genomic evolution in other settings, such as SARS-CoV-2. Also included are methods used. (DOCX) [file pone.0303257.s001.docx]

# **Supporting information – Results/Discussion**

## **Examples of established mechanisms for non-random DNM formation**

Every new generation in any species adds *de novo* single base variants to the population gene pool. Mutational ‘hot spots’ are well described[1-9], and account for increased likelihood of nucleotide variation within regions of open chromatin, GC-rich DNA, recombination (which itself is not stochastic), homopolymeric repeats, and previously mutated or gene-converted DNA sequences, among others. The overall contribution of mutation prone regions to genomic diversity, selective constraint, and impact on fitness calculations, however, has not been determined. The frequency of such DNA characteristics in essential versus non-essential genes is also unknown.

From a purely biochemical perspective, SNPs are often viewed as an assemblage of errors caused by defective base pairing or faulty proofreading of newly synthesized DNA. For example, reactive oxygen species elicit DNA lesions in the form of 8-oxo-guanine, which result in mispairing, and, if not corrected by DNA glycosylase (e.g., OGG1; functionally conserved in eukaryotes from yeast to human) generate G↔T and A↔C transversion base replacements[10, 11]. Transition mutations (adenine/guanine (A↔G) and cytosine/thymine (C↔T)) appear much more frequently in virtually every genomic setting compared to their transversional counterparts, a finding among metazoans that is commonly taken as a biochemical liability caused by CpG DNA methylation and subsequent nucleotide deamination[3, 12, 13]. Findings in the present report describe a strong and fundamental bias that underlies transition and synonymous DNMs in numerous species—which in many cases appears independent of DNA methylation—and suggests both evolutionary significance and conservation of this process (see also below).

### **Modeling genomic evolution in yeast and human**

Computer simulation was used to illustrate the extent to which purifying selection would influence non-synonymous versus synonymous SNP frequencies over time. Pronounced effects were observed at previously established human values of µ and imposed values of λ (percent of non-synonymous SNPs leading to purifying selection), either of which strongly impact the resulting SNP tabulations. For example, at µ > 0.5, the genome of any species tends towards meltdown (Fig 1). This process is accelerated dramatically as the rate of purifying selection (λ) increases. Fig 2 (top panel) depicts the relationship between species survival and synonymous SNP enhancement within evolving human populations. Findings in the figure rapidly become independent of the number of genomic replications (i.e., the process equilibrates beyond generation #10), and are also independent of genome size. For humans, where µ is ~90, ‘weeding out’ of deleterious non-synonymous SNPs and their associated genomes eliminates the entire population within a modest number of generations (~10) at high λ (>10% of non-synonymous SNPs expunged). In other words, if DNMs occur at stochastic positions, it is impossible for purifying selection to achieve the non-synonymous to synonymous SNP ratio reported by gnomAD (1.5-2.5 to 1). On the other hand, for a species such as *S. cerevisiae* with much lower µ, genomic meltdown never occurs (Fig 1), and the level of purifying selection (i.e., the magnitude of λ) required to achieve a non-synonymous to synonymous SNP ratio near the observed value of ~1 (Table 2) is never attainable (Fig 2, bottom panel).

Based on results from computer simulations and a manual step-through of expected mutations, the fraction of synonymous SNPs after eliminating a population of non-synonymous point mutations at λ can be calculated as s / (1 - (1 - s) * λ), where s is the expected fraction of synonymous SNPs if all mutations are random. Survival rate of each generation is derived from the average likelihood of any given organism surviving with the number of non-synonymous SNPs expected based on μ and a non-synonymous to synonymous SNP production ratio of ~3:1 (value derived from the genetic code for random DNMs corrected for codon usage).

Please note that yeast genomic analysis in our study provides one example of a useful model that indicates non-random SNP formation during DNA evolution. We describe reinforcing data (from our laboratory and others) showing biochemical evidence of non-random SNP accrual during DNA replication[12-15], biased SNP formation during viral pathogenesis[16], non-random SNP formation by Arabidopsis[7], similar conclusions from studies of ancient sea lamprey[17], as well as comparable non-synonymous SNP bias in bird, zebrafish, and mammalian species[18]. In addition, we describe ways in which our findings apply to well characterized (individual) human genes[19, 20], cutting edge genomic databases such as gnomAD[21], and large genomic/epidemiologic studies of de novo mutation[8, 22, 23], all of which indicate relevance of our findings to the human gene pool.

### **pLOF frequency in essential versus random or ultra-large genes (S1 Table)**

While essential genes exhibit the expected modest (~5.8-fold) decrease in pLOFs per exonic base pair compared to random genes (and ~4.1-fold decrease versus ultra-large genes), when one contrasts high levels of DNM frequency variation versus either non-synonymous SNP depletion or pLOF rates in gnomAD, the effects of ‘weeding out’ seem minimal (i.e., often only a few-fold difference for ostensibly highly constrained genes in gnomAD versus 100-fold differences that have been reported in DNM positional bias, see text). Notably, we also observed that extremely large genes (with the greatest intronic content) have statistically lower pLOF density (on a per exonic nucleotide basis) than random genes despite the fact they are not viewed as ‘indispensable’ and often exhibit a haplosufficient phenotype when studied in mice or humans (S1 Table). Whether low numbers of pLOF SNPs in ultra-large genes reflect a crucial role in the distant past—an interpretation suggested in the following section—will require further study.

**S1 Table: Average number of SNPs per exonic base pair in distinct gene categories**.

|  | Average size in base pairs (bp) | Average number of exonic base pairs (Xbp) | Synonymous SNPs (Syn) per gene | Non-synonymous SNPs (NS) per gene | pLOFs per gene | Syn/Xbp | NS/Xbp | pLOF/Xbp | Non-synonymous/  Synonymous |
| --- | --- | --- | --- | --- | --- | --- | --- | --- | --- |
| Large genes | 1,424,166 | 3362 | 264.3 | 566.4 | 14.72 | 0.08231* | 0.17180 | 0.00517** | 2.13 |
| Random genes | 82,263 | 2042 | 152.2 | 328.6 | 14.41 | 0.07375 | 0.15559 | 0.00730 | 2.14 |
| Essential genes | 58,902 | 3300 | 248 | 369 | 4.00 | 0.07699 | 0.10288*** | 0.00126*** | 1.41 |

The gnomAD site (<https://gnomad.broadinstitute.org/> ) was mined for 101 randomly selected genes, 47 of the largest genes in the human genome ((based on <http://www.cshlp.org/ghg5_all/section/gene.shtml>[24]), and 140 genes described as essential[25]. * p < .02 (compared to random genes in the same column). Note that non-synonymous to synonymous SNP ratios exhibit a modest decrease for essential genes (by ~35%) which is much smaller than marked variation in DNM rate for many genes based on direct measurement (up to 5,000% variation [8], see text). Relevant datasets are available as Supporting Information. ** p < 0.025, *** p < 0.0001 (compared to random genes in the same column) (Datasets are provided in S2 Table, Human gene datasets; see also **Supporting information – Results/Discussion**)

## **Examples showing broad implications of the evolutionary mechanism described here**

When one considers mutational meltdown and the role of non-random *de novo* SNP production, the practical ramifications can be surprising. Two speculative examples (one involving ancient adaptive biology and another modern) are provided below:

### **Species expansion during the Cambrian**

The tendency towards meltdown and biased *de novo* SNP formation bear on the significance of spliceosomal intronic DNA, which appeared prominently during the Precambrian, and before the marked evolutionary diversification of higher species (eukaryogenesis)[26, 27]. Prior to expansion of introns, one can argue that genomes with high µ faced a dilemma. While survival was dependent on a need to mutate and diversify, mutations in protein coding DNA would also inevitably meltdown at higher values of µ. It is reasonable to imagine, therefore, that spliceosomal intronic (as well as other noncoding or regulatory DNA) could have evolved—at least in part—as a means to generate prodigious variation without the need to tamper with highly conserved (and evolutionarily vital) open reading frames. Whether or not one accepts introns as having arisen (or expanded) largely for this purpose, there is no question that intronic and other non-coding (regulatory) DNA elements subserve that function in modern eukaryotic genomes. Non-coding DNA contains the majority of nucleotide differences between species, has provided a safe harbor for extensive evolutionary ‘experimentation’ required to enhance fitness, is far more abundant in higher organisms compared to protein coding elements, and constitutes the main location of genomic diversity among metazoans. The presence of introns, themselves, ensures a means to generate future diversity with far less risk of meltdown, since large numbers of SNPs placed randomly in non-coding regions of DNA are much better tolerated­ in comparison to alterations of protein coding domains. Non-coding DNA also represents a strong preferential site for DNM formation (and is anything but “neutral” or “near neutral”)[19, 28, 29]. Based on considerations such as these, we believe eukaryotic evolution has been significantly bolstered after the Precambrian by the appearance of intronic and other regulatory DNA, with introns representing a predominant means of allowing greater µ, overcoming bottlenecks, avoiding meltdown, and contributing to marked species expansion.

### **Estimating genomic evolution in other settings: do SARS-CoV-2 DNMs occur randomly?**

While the current report has focused on eukaryotic DNA, similar considerations apply to much less complex genomes, including viral pathogens that replicate in eukaryotic cells. For example, µ for SARS-CoV-2 in human tissue has been estimated as 10^-6^ mutations per genome per generation[30]. This value indicates negligible risk of meltdown, since each generation produces a marked excess of the parental (reservoir) viral genotype (Fig 1). A conservative estimate of generation time (i.e., the time interval between SARS-CoV-2 infection of a target cell and release of viral progeny) is ~10 hours, which amounts to ~900 viral replication cycles per year (or ~1,800 replication cycles in the first two years of the pandemic)[30, 31]. For a single SARS-CoV-2 virion and its genome of ~30,000 nucleotides, therefore, one can roughly predict on the order of 54 new mutations (1,800 cycles x 30,000 base pairs x 10^-6^ mutations per base pair per cycle) during the first 24 months of COVID-19. The omicron variant which appeared in pandemic year 2 contained ~50 new mutations compared to the founder strain—in surprisingly good agreement with that approximation. Whether omicron evolved in a single immunosuppressed host (as suggested by some sources[32]) or sequential infection of hundreds of human hosts is not known.

Importantly, roughly forty of fifty mutations in omicron occur in the ~3,000 base pair spike protein coding region. A virus that placed ~80% of its new mutations in roughly 10% of the genome obviously cannot be explained by random SNP formation alone. For example, computer simulations of this process and mathematical modeling (**Supporting information methods**) indicate the likelihood of omicron evolving by stochastic mutagenesis is 1 in 2.6 million trillion trillion, a value unapproachable by two years of viral evolution. Moreover, if one applies random DNMs together with robust selective pressure to account for omicron, that would require ~360 (not ~10) SNPs to have been formed in non-spike regions of the omicron genome, and that ~350 of these must have been removed by purifying selection. The establishment of 350 new (non-spike) mutations in SARS-CoV-2 at µ = 10^-6^ would require ~15 years—a far longer time period than has been available. Without either a dramatic increase of µ (punctuated adaptation) and/or marked positional bias (robust non-randomness) of viral DNMs, therefore, conventional models decidedly fail to explain omicron evolution. The notion that ~350 random mutations in non-spike RNA have been ‘weeded out’ is also problematic, since this requires 97% of random non-spike mutations must have impaired viral protein function and overall fitness (only ~10 of the expected 360 non-spike SNPs are still present in omicron). In addition, a large number of these (e.g., ~90) are expected to have been synonymous, most of which should have negligible impact. DNM enrichment of synonymous mutations in SARS-CoV-2 has been reported, and supports the notion of strongly biased SNP formation[16].

Analyses such as these further highlight shortcomings of a “random” SNP assumption as a means to address important questions regarding genomic evolution in human cells, including adaptation of eukaryotic pathogens such as SARS-CoV-2. Because clinical outcomes with existing COVID-19 drugs—including molnupiravir (which works by accelerating mutational meltdown) and ritonavir (which can be inactivated by mutations in the viral protease)—are highly dependent on features of SARS-CoV-2 evolution, the conceptual models presented here are of importance. For example, if upwards of 97% of random mutations in the protease are either: 1) much less likely to occur, or 2) in fact deleterious to the virus, that would bode well for a drug like ritonavir.

# **Supporting information methods**

## **DNA isolation and sequence analysis**

Because certain endpoints intended for evaluation of yeast mutation bias have not been available in readily accessible format (some exceptions include[33-35]), DNA sequencing was performed using standard laboratory *S. cerevisiae* (*dj MATa ilu2*). In contrast to SNP accumulation studies (which are informative over a comparatively short time period of laboratory passaging), studies of DNA strain characteristics can indicate more chronic adaptive effects requiring years of evolution.

Genomic DNA extraction/purification was performed using conventional ZR Fungal/Bacterial DNA MicroPrep™ kits (Zymo Research), with DNA provided at 500 ng/μl for library preparation and sequence analysis. A Qubit (Invitrogen) broad range fluorescent assay with fragmentation by Covaris S2 sonicator (duty cycle 10, intensity 5, cycles burst^-1^ 200 and using frequency sweep) was used to generate fragments with an average size of 300bp. The DNA was purified with Ampure Beads (Beckman-Coulter) according to manufacturer’s instructions. Following fragment purification, DNA underwent Illumina (San Diego CA) genomic DNA library preparation. DNA was blunt ended, phosphorylated and adenosine added. Branched oligonucleotide adaptors were ligated and sequencing libraries quantitated using the Kapa Biosystems kit (Woburn MA) and HiSeq2000 equipment.

SNPs were identified at high stringency (depth >100, Q score ≥ 30) for polymorphisms genome-wide, with additional verification by manual inspection using Integrative Genomics Viewer (IGV) software. SNPs were assessed manually for indicators of sequencing artifact (repeats, indels, misaligned/poor quality reads, inconsistent consensus sequence data, artifactually high coverage (‘pile up’) due to homologous sequences elsewhere in the genome, duplicated reads, etc.). In agreement with sequencing depth and Q score, SNPs evaluated in this fashion were valid as judged by direct visualization of genomic context. Complete sequence data was compared to the published reference strain ([http://downloads.yeastgenome.org/sequence/S288C_reference/ genome_releases/](http://downloads.yeastgenome.org/sequence/S288C_reference/%20genome_releases/)).

## **Computer and mathematical modeling**

The effect of negative selection on non-synonymous to synonymous SNP ratios and population viability was simulated in Python for five million evolving organisms with assignment of one SNP for each new generation per genome (µ = 1). SNPs were randomly designated as non-synonymous or synonymous based on expected ratios (corrected to codon usage), and each non-synonymous SNP checked against a value of λ (percentage of non-synonymous SNPs expunged from the gene pool). Organisms with a lethal genomic burden were removed from the population before subsequent SNPs were added, one round at a time. Average ratios of non-synonymous to synonymous SNPs among survivors were used to calculate percentage of synonymous SNPs. A similar approach was used to approximate µ = 90 or µ = 0.004, with the number of mutations each organism received determined by µ multiplied by the number of generations in which a mutation could occur.

Calculating likelihood of a COVID strain with 30,000 nucleotides in which 40 of 50 single nucleotide polymorphisms were located in the spike protein sequence was performed by placing point mutations randomly throughout the viral genome. A computer model was built to simulate establishment of mutations randomly within a set of 30,000 possible genomic positions. Fifty mutations were generated using a random number between 1 and 30,000 and placed at the corresponding locations. This process was repeated ten million times. The highest number of mutations occurring within a specific 3,000 nucleotide interval was 19, while the average number of occurrences was 4.99. As confirmation, binomial distribution was used to estimate likelihood of the known omicron SNP distribution occurring by chance. For p [probability] = 0.1, N [number of trials or instances] = 50 and X [number of successes or mutations in the desired section] ≥ 40), we found likelihood to be 3.8 x 10E-31, or 1 in 3.8 x 10E31. In other words, the likelihood of omicron evolving by chance is one in 2.6 million trillion trillion (2.6 x 10^31^), a value unapproachable by two years of viral evolution (see also **Supporting information – Results/Discussion**).

## **Statistics**

Expected values for non-synonymous:synonymous and coding:non-coding SNP frequencies in yeast were derived from codon usage (sacCer2; Codon Usage Database (<http://www.kazusa.or.jp/codon/>)), and known sequence lengths of exonic vs. intronic/intergenic DNA in *S.* *cerevisiae* ([http://downloads.yeastgenome.org/sequence /S288C_ reference/genome_releases/](http://downloads.yeastgenome.org/sequence%20/S288C_%20reference/genome_releases/)), respectively. All possible mutations in all codons (including transition versus transversion SNPs) were estimated on a stochastic basis from the genetic code and weighted according to usage. In order to maintain a robust statistical analysis, well validated tools were utilized. ‘Observed’ SNP counts were compared to ‘expected’ using chi-square analysis (2 x 2 contingency tables), which is applicable for detecting differences between measured and expected levels of DNA polymorphism. Similar analyses were applied to human DNA. To assess the strength of linear relationships between predicted loss of function (pLOF) variants per exonic base pair and non-synonymous to synonymous SNP ratios, Pearson’s correlation coefficients were calculated using <https://www.socscistatistics.com/tests/pearson/default2.aspx>. Alternative approaches such as Spearman’s correlation assess monotonic relationships between two continuous variables; however, we were interested in evaluating linear relation(s) between these parameters. A p-value below 0.05 was considered statistically significant.

**REFERENCES**

1. Chattopadhyay S, Weissman SJ, Minin VN, Russo TA, Dykhuizen DE, Sokurenko EV. High frequency of hotspot mutations in core genes of Escherichia coli due to short-term positive selection. Proc Natl Acad Sci U S A. 2009;106(30):12412-7. Epub 2009/07/21. doi: 10.1073/pnas.0906217106. PubMed PMID: 19617543; PubMed Central PMCID: PMCPMC2718352.

2. Amos W. Even small SNP clusters are non-randomly distributed: is this evidence of mutational non-independence? Proc Biol Sci. 2010;277(1686):1443-9. Epub 2010/01/15. doi: 10.1098/rspb.2009.1757. PubMed PMID: 20071383; PubMed Central PMCID: PMCPMC2871933.

3. Hodgkinson A, Ladoukakis E, Eyre-Walker A. Cryptic variation in the human mutation rate. PLoS Biol. 2009;7(2):e1000027. Epub 2009/02/06. doi: 10.1371/journal.pbio.1000027. PubMed PMID: 19192947; PubMed Central PMCID: PMCPMC2634788.

4. Francioli LC, Polak PP, Koren A, Menelaou A, Chun S, Renkens I, et al. Genome-wide patterns and properties of de novo mutations in humans. Nat Genet. 2015;47(7):822-6. Epub 2015/05/20. doi: 10.1038/ng.3292. PubMed PMID: 25985141; PubMed Central PMCID: PMCPMC4485564.

5. Garvin MR, Gharrett AJ. Evolution: are the monkeys' typewriters rigged? R Soc Open Sci. 2014;1(2):140172. Epub 2015/06/13. doi: 10.1098/rsos.140172. PubMed PMID: 26064538; PubMed Central PMCID: PMCPMC4448893.

6. Ma X, Rogacheva MV, Nishant KT, Zanders S, Bustamante CD, Alani E. Mutation hot spots in yeast caused by long-range clustering of homopolymeric sequences. Cell Rep. 2012;1(1):36-42. Epub 2012/07/27. doi: 10.1016/j.celrep.2011.10.003. PubMed PMID: 22832106; PubMed Central PMCID: PMCPMC3408629.

7. Monroe JG, Srikant T, Carbonell-Bejerano P, Becker C, Lensink M, Exposito-Alonso M, et al. Mutation bias reflects natural selection in Arabidopsis thaliana. Nature. 2022. Epub 20220112. doi: 10.1038/s41586-021-04269-6. PubMed PMID: 35022609.

8. Michaelson JJ, Shi Y, Gujral M, Zheng H, Malhotra D, Jin X, et al. Whole-genome sequencing in autism identifies hot spots for de novo germline mutation. Cell. 2012;151(7):1431-42. Epub 2012/12/25. doi: 10.1016/j.cell.2012.11.019. PubMed PMID: 23260136; PubMed Central PMCID: PMCPMC3712641.

9. Halldorsson BV, Palsson G, Stefansson OA, Jonsson H, Hardarson MT, Eggertsson HP, et al. Characterizing mutagenic effects of recombination through a sequence-level genetic map. Science. 2019;363(6425). Epub 2019/01/27. doi: 10.1126/science.aau1043. PubMed PMID: 30679340.

10. Friedberg Errol C. WGC, Siede Wolfram, Wood Richard D., Schultz Roger A., Ellenberger Tom. DNA Damage. DNA Repair and Mutagenesis. 2nd ed: Wiley Online Library; 2005. p. 9-69.

11. Singh KK, Sigala B, Sikder HA, Schwimmer C. Inactivation of Saccharomyces cerevisiae OGG1 DNA repair gene leads to an increased frequency of mitochondrial mutants. Nucleic Acids Res. 2001;29(6):1381-8. Epub 2001/03/10. doi: 10.1093/nar/29.6.1381. PubMed PMID: 11239005; PubMed Central PMCID: PMCPMC29743.

12. Fryxell KJ, Moon WJ. CpG mutation rates in the human genome are highly dependent on local GC content. Mol Biol Evol. 2005;22(3):650-8. Epub 2004/11/13. doi: 10.1093/molbev/msi043. PubMed PMID: 15537806.

13. Hernando-Herraez I, Prado-Martinez J, Garg P, Fernandez-Callejo M, Heyn H, Hvilsom C, et al. Dynamics of DNA methylation in recent human and great ape evolution. PLoS Genet. 2013;9(9):e1003763. Epub 2013/09/17. doi: 10.1371/journal.pgen.1003763. PubMed PMID: 24039605; PubMed Central PMCID: PMCPMC3764194.

14. Freeland SJ, Hurst LD. The genetic code is one in a million. J Mol Evol. 1998;47(3):238-48. Epub 1998/09/11. doi: 10.1007/pl00006381. PubMed PMID: 9732450.

15. Bofkin L, Goldman N. Variation in evolutionary processes at different codon positions. Mol Biol Evol. 2007;24(2):513-21. Epub 20061121. doi: 10.1093/molbev/msl178. PubMed PMID: 17119011.

16. De Maio N, Walker CR, Turakhia Y, Lanfear R, Corbett-Detig R, Goldman N. Mutation Rates and Selection on Synonymous Mutations in SARS-CoV-2. Genome Biol Evol. 2021;13(5). doi: 10.1093/gbe/evab087. PubMed PMID: 33895815; PubMed Central PMCID: PMCPMC8135539.

17. Cui G, Hong J, Chung-Davidson YW, Infield D, Xu X, Li J, et al. An Ancient CFTR Ortholog Informs Molecular Evolution in ABC Transporters. Dev Cell. 2019;51(4):421-30.e3. Epub 2019/11/05. doi: 10.1016/j.devcel.2019.09.017. PubMed PMID: 31679858; PubMed Central PMCID: PMCPMC7665244.

18. Wolf JB, Künstner A, Nam K, Jakobsson M, Ellegren H. Nonlinear dynamics of nonsynonymous (dN) and synonymous (dS) substitution rates affects inference of selection. Genome Biol Evol. 2009;1:308-19. Epub 2009/01/01. doi: 10.1093/gbe/evp030. PubMed PMID: 20333200; PubMed Central PMCID: PMCPMC2817425.

19. Hill AE, Plyler ZE, Tiwari H, Patki A, Tully JP, McAtee CW, et al. Longevity and plasticity of CFTR provide an argument for noncanonical SNP organization in hominid DNA. PLoS One. 2014;9(10):e109186. Epub 2014/10/29. doi: 10.1371/journal.pone.0109186. PubMed PMID: 25350658; PubMed Central PMCID: PMCPMC4211684.

20. Plyler ZE, Hill AE, McAtee CW, Cui X, Moseley LA, Sorscher EJ. SNP Formation Bias in the Murine Genome Provides Evidence for Parallel Evolution. Genome Biol Evol. 2015;7(9):2506-19. Epub 2015/08/09. doi: 10.1093/gbe/evv150. PubMed PMID: 26253317; PubMed Central PMCID: PMCPMC4607513.

21. Lek M, Karczewski KJ, Minikel EV, Samocha KE, Banks E, Fennell T, et al. Analysis of protein-coding genetic variation in 60,706 humans. Nature. 2016;536(7616):285-91. Epub 2016/08/19. doi: 10.1038/nature19057. PubMed PMID: 27535533; PubMed Central PMCID: PMCPMC5018207.

22. Iossifov I, Ronemus M, Levy D, Wang Z, Hakker I, Rosenbaum J, et al. De novo gene disruptions in children on the autistic spectrum. Neuron. 2012;74(2):285-99. doi: 10.1016/j.neuron.2012.04.009. PubMed PMID: 22542183; PubMed Central PMCID: PMCPMC3619976.

23. Besenbacher S, Sulem P, Helgason A, Helgason H, Kristjansson H, Jonasdottir A, et al. Multi-nucleotide de novo Mutations in Humans. PLoS Genet. 2016;12(11):e1006315. Epub 2016/11/16. doi: 10.1371/journal.pgen.1006315. PubMed PMID: 27846220; PubMed Central PMCID: PMCPMC5147774.

24. Scherer S. Guide to the Human Genome: CSH Press; 2010 [Gene Structure]. Available from: <http://www.cshlp.org/ghg5_all/section/gene.shtml>.

25. Bartha I, di Iulio J, Venter JC, Telenti A. Human gene essentiality. Nat Rev Genet. 2018;19(1):51-62. Epub 2017/10/31. doi: 10.1038/nrg.2017.75. PubMed PMID: 29082913.

26. Koonin EV. The origin of introns and their role in eukaryogenesis: a compromise solution to the introns-early versus introns-late debate? Biol Direct. 2006;1:22. Epub 20060814. doi: 10.1186/1745-6150-1-22. PubMed PMID: 16907971; PubMed Central PMCID: PMCPMC1570339.

27. Gilbert W, de Souza SJ, Long M. Origin of Genes. Proceedings of the National Academy of Sciences. 1997;94(15):7698-703. doi: 10.1073/pnas.94.15.7698.

28. Hong JS, Tindall Janice M., Tindall, Samuel R., Sorscher, Eric J. Mutation accumulation in *H. sapiens* F508del CFTR countermands dN/dS type genomic analysis. Submitted. 2022.

29. Ohta T, Gillespie JH. Development of Neutral and Nearly Neutral Theories. Theor Popul Biol. 1996;49(2):128-42. doi: 10.1006/tpbi.1996.0007. PubMed PMID: 8813019.

30. Bar-On YM, Flamholz A, Phillips R, Milo R. SARS-CoV-2 (COVID-19) by the numbers. Elife. 2020;9. Epub 20200402. doi: 10.7554/eLife.57309. PubMed PMID: 32228860; PubMed Central PMCID: PMCPMC7224694.

31. Sender R, Bar-On YM, Gleizer S, Bernshtein B, Flamholz A, Phillips R, et al. The total number and mass of SARS-CoV-2 virions. Proc Natl Acad Sci U S A. 2021;118(25). doi: 10.1073/pnas.2024815118. PubMed PMID: 34083352; PubMed Central PMCID: PMCPMC8237675.

32. Wei C, Shan KJ, Wang W, Zhang S, Huan Q, Qian W. Evidence for a mouse origin of the SARS-CoV-2 Omicron variant. J Genet Genomics. 2021;48(12):1111-21. Epub 20211224. doi: 10.1016/j.jgg.2021.12.003. PubMed PMID: 34954396; PubMed Central PMCID: PMCPMC8702434.

33. Liu H, Zhang J. Yeast Spontaneous Mutation Rate and Spectrum Vary with Environment. Curr Biol. 2019;29(10):1584-91.e3. Epub 20190502. doi: 10.1016/j.cub.2019.03.054. PubMed PMID: 31056389; PubMed Central PMCID: PMCPMC6529271.

34. Peter J, De Chiara M, Friedrich A, Yue JX, Pflieger D, Bergström A, et al. Genome evolution across 1,011 Saccharomyces cerevisiae isolates. Nature. 2018;556(7701):339-44. Epub 2018/04/13. doi: 10.1038/s41586-018-0030-5. PubMed PMID: 29643504; PubMed Central PMCID: PMCPMC6784862.

35. Zhu YO, Siegal ML, Hall DW, Petrov DA. Precise estimates of mutation rate and spectrum in yeast. Proc Natl Acad Sci U S A. 2014;111(22):E2310-8. Epub 2014/05/23. doi: 10.1073/pnas.1323011111. PubMed PMID: 24847077; PubMed Central PMCID: PMCPMC4050626.
